# Association between Plasma Trimethylamine N-Oxide Levels and Type 2 Diabetes: A Case Control Study

**DOI:** 10.3390/nu14102093

**Published:** 2022-05-17

**Authors:** Nora A. Kalagi, Rohith N. Thota, Elizabeth Stojanovski, Khalid A. Alburikan, Manohar L. Garg

**Affiliations:** 1 Nutraceuticals Research Program, School of Biomedical Sciences and Pharmacy, University of Newcastle, Callaghan, NSW 2308, Australia; nora.kalagi@uon.edu.au (N.A.K.); rohith.thota@newcastle.edu.au (R.N.T.); 2 Department of Clinical Pharmacy, College of Pharmacy, King Saud University, Riyadh 11362, Saudi Arabia; kalburikan@ksu.edu.sa; 3 Department of Biomedical Sciences, Macquarie University, Sydney, NSW 2109, Australia; 4 School of Mathematical and Physical Science, University of Newcastle, Callaghan, NSW 2308, Australia; elizabeth.stojanovski@newcastle.edu.au

**Keywords:** trimethylamine N-oxide, Type 2 diabetes, diet, case control study

## Abstract

Animal and human studies have reported conflicting results on the relationship between circulating trimethylamine N-oxide (TMAO) levels and risk of Type 2 diabetes (T2D). This study aimed to compare plasma TMAO levels in people with or without T2D and explore the association of TMAO and T2D. A prospective case-control study of 297 participants, 164 healthy controls and 133 patients with T2D, was conducted. TMAO levels were quantified by UPLC-MS/MS. Comorbidities, dietary patterns, physical activity, and blood biomarkers were assessed. Median (IQR) plasma TMAO levels were significantly higher in diabetes cases (4.95 (2.84–8.35) µmol/L) compared to healthy controls (3.07 (2.05–4.82) µmol/L) (*p* < 0.001). The association between TMAO and T2D was significant in the non-adjusted Model 1 (*p* < 0.001) and after adjusting for confounders of diabetes including age, BMI, and level of education in Model 2 (*p* = 0.04). When the association was further adjusted for physical activity and diet in Model 3, plasma TMAO levels at only the highest quartile (>6.40 µmol/L) were associated with the risk of diabetes (OR = 3.36, 95% CI [1.26, 9.04], *p* = 0.02). The results presented suggest an association between plasma TMAO levels and T2D. A significant correlation was found between red meat consumption and increased levels of TMAO in T2D patients. A longitudinal study is warranted to further evaluate the correlation between TMAO and T2D.

## 1. Introduction

Recent studies have provided evidence for the involvement of gut microbiota in the development of cardiovascular disease (CVD) and complicated metabolic diseases, including obesity and Type 2 diabetes (T2D) [1,2]. Trimethylamine N-oxide (TMAO) is a metabolite produced in the gut microbiota as a byproduct of the digestion of dietary compounds such as choline/phosphatidylcholine and l-carnitine and degraded into trimethylamine (TMA) which is absorbed in the circulation and oxidized in the liver by the flavin monooxygenase (FMO3) enzyme [1]. Large deviation in TMAO levels can be attributed to dietary differences and gut microbial composition, both of which play an important role in TMAO level variation. Other factors, such as kidney disease and advancing age, have been reported to influence TMAO levels, complicating its association with disease aetiology [3,4].

High consumption of red meat, eggs, and dairy products has been linked to elevated TMAO levels in the blood [2,5] and independently associated with an increased risk of T2D [6,7]. In animal models, TMAO has been shown to induce T2D via increasing fasting insulin levels and insulin resistance (HOMA-IR), aggravating impaired glucose tolerance [8,9], inducing adipose tissue inflammation, and obstructing the hepatic insulin signalling pathway [8]. Studies in humans have shown that high TMAO levels are associated with an increased risk of T2D [9,10,11,12,13,14]. Cardiac patients with elevated TMAO levels are more likely to develop diabetes, suggesting age and body mass index (BMI) to be the major determinants for TMAO levels [15]. Although previous evidence has suggested the association of TMAO with T2D pathogenesis, the precise mechanisms remain unclear.

Type 2 diabetes has been prevalent in Saudi Arabia (SA) in the last twenty years [16]. According to the International Diabetes Federation (IDF) Atlas report for 2019, SA is ranked fourth among the Middle East and North African (MENA) countries in relative to the number of people with diabetes mellitus [17]. Socioeconomic factors and lifestyle behaviours, including unhealthy diet, lack of physical activity, smoking, and obesity, were the primary triggers for the increased incidence of T2D in SA [16,18]. Despite the efforts undertaken by the government to control the increase in the disease prevalence rate and its complications, it would seem challenging with the advances in aging, population growth, and unhealthy habits of the community [19]. This study aimed to compare the TMAO levels in people with or without Type 2 diabetes and explore the association of TMAO and Type 2 diabetes in a Saudi Arabian cohort.

## 2. Materials and Methods

### 2.1. Study Design and Population

This study was a single-centre, prospective case-control study conducted between December 2018 and March 2020 at King Saud University Medical City (KSUMC) in Riyadh, Saudi Arabia. Blood samples and clinical data were collected from participants from Saudi Arabian nationals who had a clinician confirmed diagnosis of Type 2 diabetes mellitus (cases, *n* = 133) or had no history of Type 2 diabetes mellitus (controls, *n* = 164). Diagnosis of Type 2 diabetes were based on the American Diabetes Association (ADA) and the European Association for the Study of Diabetes (EASD) criteria: fasting plasma glucose (FPG) ≥7 mmol/L, haemoglobin A1c (HbA1c) ≥6.5%, and random blood sugar ≥11.1 mmol/L [20]. They were aged between 20 and 75 years old and had not participated in any clinical trial for at least three months. Pregnant and breast-feeding women, participants with a history of severe neurological diseases or seizures, currently on any antimicrobials (antibacterial, antifungal, antiviral) known to influence the gut microbiome, on haemodialysis or peritoneal dialysis, history of the new investigational drug three months prior to this trial, and unwilling to provide informed consent, were excluded. A flowchart of study participants’ screening and quantification is provided (Supplementary Figure S1). Written informed consent was obtained from all study participants before participating in the study. The study protocol was approved by the University of Newcastle Human Research Ethics Committee (H-2018-0138) and the King Saud University Institutional Review Board (E-18-3073).

### 2.2. Clinical Data

After the screening, demographic-related information and medical history were obtained from all eligible participants via a self-administered questionnaire, including gender, age, level of education, other comorbidities, and lifestyle habits, such as smoking status and allergies. Medication history included all the medications and supplements the participants were taking. Anthropometric measurements including height and weight were also measured. Height was measured using a wall-mounted stadiometer, and weight was measured in kilograms using calibrated digital scales.

### 2.3. Assessment of Physical Activity and Dietary Patterns

#### 2.3.1. International Physical Activity Questionnaire Short Version

The participants’ physical activity was assessed using the official Arabic short-form of the International Physical Activity Questionnaire (IPAQ) available online at www.ipaq.ki.se (accessed on 1 December 2021) [21]. The IPAQ short-form has seven questions that gather information on the time spent by participants in vigorous and moderate-intensity physical activities and walking, including frequency (days per week) and duration (minutes per day) of at least ten minutes in the previous seven days. The questionnaire also measured sedentary time in minutes per day.

The total level of physical activity was determined by multiplying the MET intensity for each activity by the minutes/week engaged in each activity to obtain the MET-score (metabolic equivalents per minute/week). IPAQ was scored using MET intensities of 8 METs for vigorous exercise, 4 METs for moderate activity, and 3.3 METs for walking. Participants were categorized as low, moderate, and high physical activity levels based on the summation of the MET-score calculated for each activity. Data from the IPAQ were processed according to the IPAQ authors’ guideline [22].

#### 2.3.2. Food Frequency Questionnaire: Saudi Version

The validated Saudi version of the Food Frequency Questionnaire (FFQ) was used to assess participants’ dietary patterns [23]. The questionnaire included a list of 140 different food items, categorized into nine food groups, including meats, bread and cereals, sandwiches and burgers, dairy products, sweets and snacks, drinks, fruits, and vegetables. Participants were asked how frequently they had consumed each type of food item over the past year. The questionnaire responses were ranging from never or less than once per month to more than six times per day. Questions about other food products consumed by participants that were not specified in the questionnaire were also available, including the type of fat used in cooking, and the consumption of salt and dietary supplements. This questionnaire does not account for the amount of each food item consumed in serving size. Food consumption frequency was measured quantitatively by converting the frequency of each food into a score equivalent to weekly intake instead of monthly or daily consumption, based on the Victorian Cancer Council method for FFQ analysis [24].

Responses of never or once per month were given a score of zero, while food items consumed more than seven days per week were given a score of 7. In addition, food consumption frequencies were measured qualitatively by determining the frequency of several food items among patients with or without Type 2 diabetes.

### 2.4. Laboratory Measurement

Fasting blood samples were collected from all study participants for the determination of fasting plasma glucose (FPG), total cholesterol (TC), triglycerides (TG), high-density lipoprotein (HDL), low-density lipoprotein (LDL), haemoglobin A1C (HbA1C), and inflammatory markers, including high-sensitivity C-reactive protein (hs-CRP), by routine assays at the central laboratory of KSUMC, following standard laboratory protocols. For the measurement of TMAO, a separate EDTA tube of 2 mL blood was collected. Blood samples were centrifuged at 10,000× *g* for 10 min under 4 °C, and plasma was stored under −80 °C until the time of the analysis.

### 2.5. Quantification of Plasma Trimethylamine N-Oxide (TMAO) Levels

Trimethylamine N-oxide levels in plasma were quantified using ultra-high-performance liquid chromatography-tandem mass spectrometry (UPLC/MS/MS) by (Waters Corporation, Milford, MA, USA). Stable isotope d9-(trimethyl)-labelled was used as an internal standard as previously reported [25]. Briefly, frozen plasma samples were thawed, centrifuged at 5500× *g*, and kept at 4 °C for 5 min. A total of 100 µL of the plasma was used for the assay. The internal standard working solution (precipitation agent) contained 10 µmol/L of d9-TMAO produced in a mixture of methanol:acetonitrile (15:85 *v*/*v*) and 0.2% formic acid. Protein precipitation was achieved by adding 300 µL of the internal standard working mixture to 100 µL of water to build the calibration curve and quality control samples, as well as 100 µL of unknown concentration plasma. After gentle vortexing and centrifugation at 10,000× *g* for 10 min, the supernatant was injected into UPLC/MS/MS. Chromatographic separation was performed using an Acquity UPLC BEH HILIC column (100 mm × 2.1 mm, 1.7 µm particle size). TMAO and d9-(trimethyl) detection was performed by triple quadrupole mass spectrometer, using positive electrospray ionization mode with multiple reaction monitoring (MRM) of parent and daughter ion transitions: *m*/*z* 76.1→58.2 and 85.04→66.04, respectively (Supplementary Figure S2).

### 2.6. Statistical Analysis

Participant characteristics were summarized according to diabetes cases and controls as number (percentage) for categorical data and mean ± standard deviation (SD) or median (interquartile range (IQR) for continuous data. The normality assumptions of the data were verified using the Shapiro-Wilk test and histograms. Continuous variables were compared between two groups using *t*-test or Mann-Whitney U-test when appropriate. Categorical variables were compared using chi-square tests and Fisher exact test. One-way ANOVA and Kruskal-Wallis test were used to compare the participants’ characteristics among different plasma TMAO quartiles, Q1 (<2.37 µmol/L), Q2 (2.38–3.58 µmol/L), Q3 (3.59–6.39 µmol/L), and Q4 (>6.40 µmol/L). Multivariate analysis was used to assess the relationship between plasma TMAO levels, biochemical data (e.g., fasting glucose, HbA1c, lipids, physical activity, and dietary score), and other variables, including age, BMI, and level of education. Binary logistic regression was used to assess the association between TMAO and Type 2 diabetes and adjusted for confounders including age, gender, BMI, level of education, physical activity, and dietary score using a stepwise approach. All data analyses were carried out using SPSS (version 27, SPSS Inc., Chicago, IL, USA), with the *p*-value of < 0.05, indicating statistical significance.

## 3. Results

### 3.1. Baseline Characteristics

The baseline characteristics of study participants (Type 2 diabetes, *n* = 133, and healthy controls, *n* = 164) are detailed in Table 1. In comparison to the healthy controls, people with diabetes (cases) were older (55 versus 37 years, *p* < 0.001), a greater proportion of males (20.3% versus 6.7%, *p* < 0.001), had higher BMI (30.98 versus 28.04 kg/m^2^, *p* < 0.001), were regularly taking anti-hyperglycaemic (100% versus 4.9%, *p* < 0.001), lipid-lowering (79.8% versus 9.4%, *p* < 0.001), and antacid (37.6% versus 8.0%, *p* < 0.001) medications, and had lower proportion with tertiary education (39.8% versus 79.9%, *p* < 0.001). No significant differences were observed between the healthy controls and diabetes cases in smoking status and hs-CRP levels. However, significant differences between the two groups were noted in laboratory parameters including FPG, HbA1c, TC, LDL, HDL, and TG.

### 3.2. Physical Activity and Dietary Patterns

The study participants’ engagement in physical activity during the previous seven days is summarized in Table 1. People with Type 2 diabetes participated significantly less in physical activity than healthy individuals (*p* = 0.008). Of the total participants with Type 2 diabetes (*n* = 133), 6 (4.5%) and 41 (30.8%) participated in ≥3 days vigorous activities for more than 20 min/days, and ≥5 days of moderate/walking activities of more than 30 min/days. Thirty-seven participants with Type 2 diabetes (39.8%) scored a moderate level of physical activity, equivalent to 30 min of moderate-intensity physical activity on most days. Achieving a high level of physical activity was reported by only seven participants with Type 2 diabetes (5.2%), which approximates one hour of activity per day or more of moderate-intensity activity level. A significant difference was found between the MET-categories of physical activities in diabetes cases and healthy controls, *p* = 0.025. Among TMAO quartiles, no significant differences were noted in physical activity MET-score and MET-category (Table 2).

The frequencies of food items consumed among healthy controls and Type 2 diabetes cases are summarized in Supplementary Table S1. Within the meat food group, chicken and meat kabsa (a meal consisting of meat, rice, and spices) were the highest consumed food items among both study cohorts. People with Type 2 diabetes (cases) ate more meat (chicken, lamb, or camel) than healthy controls. Chicken and lamb meat stews (a meal consisting of cuts of meat, broth, and vegetables) are consumed more frequently by Type 2 diabetes compared with non-diabetes group (55.6% versus 29.3%, *p* < 0.001), and (53.4%, versus 28.6%, *p* < 0.001), respectively, up to seven days per week. Type 2 diabetes cohorts also consumed a lot of eggs and low-fat dairy products such as milk, yogurt, Laban (watered down yogurt), and white cheese. However, we observed a low frequency of consumption of carbohydrate-rich foods such as wheat and white bread and white rice, but not boiled potatoes. Diabetes patients consumed fewer calories from foods with high caloric values such as sugar, nuts, and dates, as well as fried chicken and burgers, than healthy controls. Both study participants drank a lot of black tea and Arabic coffee. It is worth noting that the Type 2 diabetes cohort consumes high amounts of seasonal fruits and vegetables. Strawberries, dried fruits, and salads, on the other hand, were more common among the healthy controls. Furthermore, we observed higher consumption of margarine and meat fat in patients with Type 2 diabetes than healthy controls. The sum of each food group’s score was also compared between the two study groups, including meat, dairy products, fibres, and eggs. The highest scores in the meat group were red meat, with significant differences of *p* = 0.042. Eggs, dairy products, and fibres were also consumed more frequently amongst Type 2 diabetes cases.

### 3.3. TMAO Levels and Biochemical Parameters

**Figure 1:**
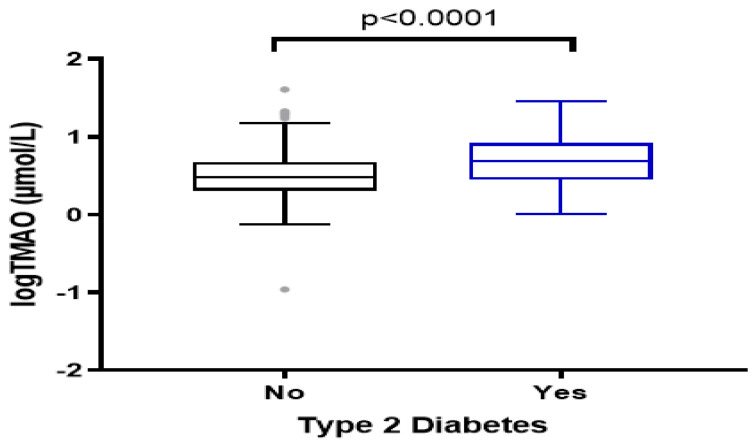
Plasma levels of TMAO among the study groups, Type 2 diabetes cases and healthy controls. The data were log−transformed for normality.

Median (IQR) of the plasma TMAO levels were significantly higher in diabetes cases (4.95 (2.84–8.35) µmol/L) compared to healthy controls (3.07 (2.05–4.82) µmol/L) (*p* < 0.001) (Figure 1). Unadjusted analyses showed that 40% of Type 2 diabetes patients were in the highest quartiles of TMAO (median >6.40 µmol/L) (Table 2). These participants were more likely to be older, have a lower level of education, and be taking glucose- and lipid-lowering medications. In addition, they have a higher HbA1c level and higher dietary intake of red meat (*p* = 0.014). No significant differences were noted in BMI, LDL, HDL, and hs-CRP levels across TMAO quartiles.

#### 3.3.1. Correlations between TMAO Levels and Markers of Metabolic Health

Multivariate analysis showed that plasma TMAO levels were positively correlated with age (r = 0.322, *p* < 0.001), fasting plasma glucose (r = 0.131, *p* = 0.024), HbA1c (r = 0.227, *p* < 0.001), and triglycerides (r = 0.115, *p* = 0.048) (Table 3). A borderline correlation was found with duration of diabetes (r = 163, *p* = 0.061) and eggs score (r = 0.1, *p* = 0.068). Correlation between plasma TMAO levels and total meat score (r = 0.106, *p* = 0.068) and fibre score (r = 0.020, *p* = 0.736) was not significant; however, a significant positive relationship was found with red meat (r = 0.130, *p* = 0.025) and dairy products score (r = 0.129, *p* = 0.026). A positive correlation was also evident between plasma TMAO levels, red meat (r = 0.121, *p* = 0.037), and eggs (r = 0.118, *p* = 0.041) consumption at a consumption frequency of more than three times per week.

#### 3.3.2. Plasma TMAO Levels and Type 2 Diabetes

As presented in Table 4, the association between TMAO as a continuous variable and Type 2 diabetes was significant in non-adjusted Model 1 (OR = 1.11, 95% CI [1.05, 1.17], *p* ≤ 0.001, and Model 2 adjusted for confounders including age, BMI, and level of education (OR = 0.06, 95% CI [1.00, 1.14], *p* = 0.04). However, in Model 3, with further adjustment for physical activity MET-score and dietary score (red meat and eggs), the association was lost (OR = 1.05, 95% CI [0.98, 1.13], *p* = 0.20). Unadjusted estimates revealed a significant association between plasma TMAO levels and T2D, with an 11% increase in the odds of having T2D by one unit increase in plasma TMAO level.

On the other hand, the association of TMAO as a categorical variable showed that participants in the quartiles with the highest TMAO level (>6.40 µmol/L) have an increased risk of diabetes in non-adjusted Model 1 (OR = 6.19, 95% CI [3.05, 12.57], *p* < 0.001); Model 2 (OR = 2.81, 95% CI [1.09, 7.27], *p* = 0.03); and Model 3 (OR = 3.36, 95% CI [1.26, 9.04], *p* = 0.02) in comparison with the lowest quartiles.

## 4. Discussion

This case-control study explored the association between plasma TMAO levels and prevalent Type 2 diabetes. Higher plasma levels of TMAO were evident in patients with T2D than in healthy controls. Circulating TMAO levels were also significantly correlated with fasting plasma glucose, HbA1c, and plasma triglycerides. However, no significant observations were found between plasma TMAO and other lipid-based parameters (total cholesterol, LDL-cholesterol) and hs-CRP. In an unadjusted model, and after adjusting for demographic variables, including age, BMI, and level of education, regression analysis showed that elevated plasma TMAO levels are linked to increased risk of T2D. However, when the analysis was further adjusted for physical activity MET-score and diet, plasma TMAO levels at only the highest quartile (>6.40 µmol/L) were associated with the risk of diabetes. The trend of association between TMAO quartiles and Type 2 diabetes revealed a strong statistically significant association. Participants in the highest quartile group (>6.4µmol/L) presented poor metabolic profile, lower education status, high medications usage, and increased consumption of red meat compared to the other quartiles.

Evidence to date for the association between elevated plasma TMAO levels and the risk of T2D is limited and has been inconsistent. Consistent with our findings, previous studies on animals and humans have revealed that plasma TMAO levels are associated with prevalent diabetes [9,10,11,12,13,14]; however, three other studies reported contradictory results [26,27,28]. The association between TMAO and Type 2 diabetes was proposed based on multiple mechanisms, including elevated fasting insulin levels and HOMA-IR, aggravating impaired glucose tolerance, and promoting adipose tissue inflammation [8]. The exacerbation in impaired glucose tolerance was probably triggered by TMAO-aggravated blockage of the insulin signalling cascade. TMAO was also associated with genes involved in the insulin signalling pathway, which stimulated obstruction of liver glycogen synthesis, promoted gluconeogenesis, and reduced hepatic glycogen transport capacity [8]. Another possible explanation for the link between TMAO and T2D is through the polymorphism of FMO3 gene [29]. Previous research has found that the link between plasma TMAO concentration and cardiovascular disease risk is more significant in patients with Type 2 diabetes than in healthy controls, implying that TMAO has a deleterious impact on Type 2 diabetes prognosis [6]. However, further studies are required to establish a precise mechanism mediating the association between circulating TMAO levels and T2D.

The current study found a significant correlation between plasma TMAO levels and FPG and HbA1c. However, these results are in contrast with other cross-sectional [30] and longitudinal studies [27]. Although median plasma TMAO levels were similar between these studies and our study, discrepancies between the study results might arise from the ethnicity of the study population, diagnosis of diabetes (self-reported vs. clinician confirmation), and method used to determine plasma TMAO (HPLC-MS vs. UPLC/MS/MS). The association between plasma TMAO and T2D is specific to the investigated cohorts, ethnicity being the biggest discrepancy factor between these studies. Plasma TMAO levels in US populations [10,27,31] differed from those in Chinese [32,33] and European studies [34]. This is the first study to report TMAO levels in an Arabic population to the best of our knowledge. The circulating TMAO in the studied Arabic population were comparable to previously reported median values for the US population [10,27,31], but not for the studies with the Chinese population [32,33]. Standardisation of the measurement of plasma TMAO (also including similar sample processing and storage for the stability of TMAO) might be a critical step in determining the ethnicity-based differences in plasma TMAO levels.

The variation in the median TMAO levels between the study cohorts could also be attributed to confounding factors, such as age, dietary habits, and host gut microbiome composition. The median age range of the population varied across studies. Similar to other studies, median age range for the diabetes group was significantly higher than the control group. Despite that, the association between TMAO and T2D remained significant after adjusting for age in the crude multivariate analysis. Age-dependent (particularly between 40–60 y) increase in TMAO levels was reported in the previous studies [35], supporting the significant effect of ageing on plasma TMAO levels observed in this study. In the current study, age was an important confounder and a determinant of T2D compared to plasma TMAO. When the association was adjusted for different quartiles, TMAO levels in the highest quartile (>6.40 µmol/L) remained a significant determinant of Type 2 diabetes. We discovered that younger age is a significant confounder in this association. People under the age of 59 had a non-significant association with TMAO, while the association was significant in those over 60 years of age. This finding was consistent with previous studies reporting the link between T2D and TMAO levels, highly confounded by younger age [30]. These patients are less likely to develop T2D in the early stages. Further studies evaluating plasma TMAO and T2D will need to control for age in the study population to reduce confounding effects of ageing on plasma TMAO for the metabolic outcomes.

Diet may also explain the variation in median plasma TMAO levels between studies. Geographical location and culturally specific factors influence population dietary habits. It has been proposed that dietary nutrients have a modulatory effect on the gut-dependent production of TMAO [36]. Given that higher TMAO production could be caused by a high intake of choline and L-carnitine, our findings were consistent with previous findings suggesting an independent association between Westernised diet and Type 2 diabetes risk [37]; however, this study did not investigate the direct relationship between TMAO and T2D risk. Red meat, which is abundant in choline and L-carnitine, was associated with higher levels of TMAO in plasma [38]. It has been demonstrated that the postprandial TMAO levels are increased in healthy young men after a high-fat diet [39]. Animal studies have reported high serum levels of TMAO in mice fed with fish protein [40], this finding supported by human studies [41]. However, our study could not confirm the link between TMAO and seafood consumption. Red meat is the only dietary component correlated with plasma TMAO levels. Nutritional habits in Saudi Arabia are remarkably comparable to the Western diet, being high in sugars, saturated fats, eggs, and animal proteins [42]. These findings could justify the similarity of median plasma TMAO levels in the US population and support the influence of dietary nutrients on TMAO levels.

Recent studies have revealed variability in the gut microbiome composition in Type 2 diabetes patients compared to healthy individuals. Patients with T2D were presented with disruption in gut microbiota homeostasis accompanied by a breakdown of the intestinal barrier integrity and an increase in epithelial permeability to influence the aberrant generation and absorption of TMAO [43]. TMA precursors such as l-carnitine and choline have been shown to modulate the gut microbiome and enhance the relative species abundance of TMA-producing bacteria in animal studies [44]. Increased TMAO was also triggered by dietary factors, including unhealthy diet, especially those high in animal fats [41].

Current study results also revealed a relationship between plasma TMAO and education level which is consistent with a previously published study [45]. Limited studies have examined the relationship between educational achievement and diabetes prevalence, reporting an inverse association in the US population [46]. However, this association may vary by race/ethnicity and gender [47]. Notably, more “well-educated” people fall in the healthy (non-diabetic) group, representing younger subjects. Higher education level may promote consumption of more healthful diets as better-educated people may access better nutrition information. Lower level of education has been shown to be a predictor of poorer dietary patterns [48].

The strength of our study is being the first to report plasma TMAO levels and determine association with the prevalent T2D in an Arabic population. We reduced intra-individual variation in the study cohort by including participants, both healthy and with Type 2 diabetes, who lived in the same geographical area and were provided with medical care from the same medical institution. We included only clinician-confirmed diagnosed people with T2D. Moreover, we excluded people taking antibiotics to avoid their influence on the gut microbiota and TMAO production [49]. Complete information about the participants’ diet and physical activity was obtained from all participants. Furthermore, in this study we used UPLC-MS/MS assay for quantification of TMAO levels.

The inequality between the study groups in terms of age and gender distribution remains a limitation of this study. The diversity in age range among the two groups might confound the association between TMAO and Type 2 diabetes in the adjusted model; therefore, age matching is suggested in future studies. Second, because of the case-control design of this study, we were unable to infer any causation between plasma TMAO levels and Type 2 diabetes. Data in the current study do not delineate if elevated plasma TMAO levels are a cause or consequence of T2D. However, we better understood the predicting factors influencing this association. Therefore, longitudinal studies are essential to predict the prognostic effect of TMAO on the development of Type 2 diabetes. Third, residual confounding could not be ruled out, even after adjusting for several potential confounders. Finally, we included only the central area and one medical centre in Riyadh; therefore, the results cannot be generalised to other populations.

In conclusion, our study has further expanded our knowledge on the relationship between circulating TMAO and Type 2 diabetes. We reported association between plasma TMAO levels and markers of Type 2 diabetes (fasting plasma glucose, HbA1c) in the Saudi Arabian population. Diet was associated with increased levels of TMAO in patients with Type 2 diabetes, specifically red meat. Longitudinal studies are warranted to elucidate the potential mechanisms between plasma TMAO and the risk of diabetes.

## Figures and Tables

**Table 1:** Baseline characteristics and metabolic parameters of subjects with or without Type 2 diabetes.

	Type 2 Diabetes		
	No (*n* = 164)	Yes (*n* = 133)	*p*-Value ^d^
**Demographics**			
Age (years) ^a^	37 (33–45)	55 (50–62)	<0.001
Gender, No. (%)			<0.001
Male	11 (6.7)	27 (20.3)	
Female	153 (93.3)	106 (79.7)	
Level of Education, No. (%)			<0.001
Primary	5 (3)	38 (28.6)	
Secondary	28 (17.1)	42 (31.6)	
Tertiary	131 (79.9)	53 (39.8)	
BMI (kg/m^2^) ^a^	28.04 (24.68–31.31)	30.98 (28.31–35.10)	<0.001
Smoking (yes), No. (%)	11(6.7)	12(9)	NS
Diabetes duration (years)	0	12 (7–20)	<0.001
Cardiovascular disease, (yes), No. (%)	0	10 (7.5)	<0.001
Gastrointestinal disease, (yes), No. (%)	5 (3)	5 (3.8)	NS
Medication use (yes), No. (%)			
Antihyperglycemic	8 (4.9)	133 (100)	<0.001
Antihyperlipidemic	16 (9.8)	104 (79.4)	<0.001
Antacids	13 (8)	50 (37.6)	<0.001
Supplement use, No. (%)			0.003
0–3	154 (93.9)	130 (97.8)	
>4	10 (6.1)	3 (2.3)	
**Laboratory Parameters**			
FPG, mmol/L ^a^	4.78 (4.44–5.14)	8.00 (6.12–10.12)	<0.001
HbA1c, % ^a^	5.45 (5.20–5.80)	8.30 (7.30–9.50)	<0.001
Total Cholesterol, mmol/L ^b^	5.15 ± 0.95	4.42 ± 1.19	<0.001
LDL-C, mmol/L ^a^	2.84 (2.34–3.39)	2.31 (1.87–2.93)	<0.001
HDL-C, mmol/L ^a^	1.53 (1.32–1.83)	1.26 (1.12–1.51)	<0.001
Triglycerides, mmol/L ^a^	1.00 (0.75–1.42)	1.34 (0.97–1.90)	<0.001
hs-CRP, mg/L ^a^	1.82 (1.11–6.20)	3.40 (1.21–7.77)	NS
**Physical Activity** ^a^			
Total MET-Score (MET-min/wk.)	443.3 (0–1017.3)	297.0 (49.5–742.0)	0.008
Vigorous MET-min/wk.	0 (0–4800) ^c^	0 (0–3840)	0.001
Moderate MET-min/wk.	0 (0–2400) ^c^	0 (0–2400)	0.001
Walking MET-min/wk.	255.8 (0–594.0)	247.5 (0–544.5)	NS
Total MET-Category, No. (%)			0.025
Low	91 (55.5)	89 (66.9)	
Moderate	56 (34.1)	37 (27.8)	
High	17 (10.4)	7 (5.2)	

BMI: body mass index; FPG, fasting plasma glucose; HbA1c, glycated haemoglobin; LDL-C, low-density lipoprotein cholesterol; HDL, high-density lipoprotein; hs-CRP, high-sensitivity C-reactive protein; TMAO, trimethylamine n-oxide, MET-min/wk., metabolic equivalent minutes per week. ^a^ Data are presented as median (IQR (25th–75th)). Mann-Whitney U-test was used in the comparison. ^b^ Data are presented as mean ± standard deviation. Student’s *t*-test was used in the comparison. ^c^ This range represents the (min–max) because the IQR was zero. ^d^ Statistically significant association (*p* < 0.05), NS: not statistically significant correlation (*p* > 0.05).

**Table 2:** Participant characteristics by quartiles of trimethylamine N-oxide (TMAO).

		Quartiles of TMAO				
	*n*	Q1 (*n* = 75)	Q2 (*n* = 73)	Q3 (*n* = 74)	Q4 (*n* = 75)	*p*-Value ^c^
TMAO, µmol/L	297	<2.37	2.38–3.58	3.59–6.39	>6.40	
**Demographics**						
Age (years) ^a^	297	40 (33–49)	41 (35–53)	49 (37–55) ^4^	55 (47–63) ^1,2,3^	<0.001
Gender, No. (%)						0.025
Male	38	4 (10.5)	12 (31.6)	7 (18.4)	15 (39.5)	
Female	259	71 (27.4)	61 (23.6)	67 (25.9)	60 (23.2)	
Level of Education, No. (%)						0.037
Primary	43	6 (14)	8 (18.6)	10 (23.3)	19 (44.2)	
Secondary	70	18 (25.7)	13 (18.6)	20 (28.6)	19 (27.1)	
Tertiary	184	51 (27.7)	52 (28.3)	44 (23.9)	37 (20.1) ^1^	
BMI (kg/m^2^) ^a^	297	29.57 (25–32.54)	28.63 (25.26–33.78)	29.31 (27.18–34.16) ^4^	30.06 (25.85–33.98) ^1^	NS
Smoking (yes), No. (%)	23	5 (21.7)	7 (30.4)	5 (21.7)	6 (26.1)	NS
Type 2 Diabetes Mellitus, No. (%)						<0.001
No	164	53 (32.3)	46 (28)	44 (26.8)	21 (12.8)	
Yes	133	22 (16.5)	27 (20.3)	30 (22.6)	54 (40.6)	
Cardiovascular disease, No. (%)						NS
No	287	74 (25.8)	70 (24.4)	73 (25.4)	70 (24.4)	
Yes	10	1 (10)	3 (30)	1 (10)	5 (50)	
Gastrointestinal disease, No. (%)						NS
No	287	74 (25.8)	71 (24.7)	73 (25.4)	69 (24)	
Yes	10	1 (10)	2 (20)	1 (10)	6 (60)	
Medication use, No. (%)						
Antihyperglycemic, (yes)	141	23 (16.3)	30 (21.3)	31 (22)	57 (40.4)	<0.001
Antihyperlipidemic, (yes)	120	20 (16.7)	24 (20)	31 (25.8)	45 (37.5)	<0.001
Antacids, (yes)	63	7 (11.1)	11 (17.5)	22 (34.9)	23 (36.5)	<0.001
Supplement use, No. (%)						NS
0–3	284	72 (25.4)	72 (25.4)	68 (23.9)	72 (25.4)	
>4	13	3 (23.1)	1 (7.7)	6 (46.2)	3 (23.1)	
**Laboratory Parameters**						
FPG, mmol/L ^a^	297	4.92 (4.50–7.72)	5.17 (4.76–6.79)	5.26 (4.62–8)	6.04 (4.68–9.08)	NS
HBA1c, % ^a^		5.70 (5.30–7.40)	5.70 (5.40–7.20)	6 (5.50–8.10)	7.30 (6–8.90) ^1,2,3^	<0.001
Total Cholesterol, mmol/L ^b^		4.72 ± 0.93	4.74 ±1.05	4.75 ± 0.93	4.81± 1.02	NS
LDL-C, mmol/L ^a^		2.63 (2.23–3.33)	2.61 (2.19–3.30)	2.49 (2.12–3.18)	2.45 (2.05—3.24)	NS
HDL-C, mmol/L ^a^		1.40 (1.18–1.69)	1.47 (119–1.72)	1.46 (1.21–1.73)	1.44 (1.19–1.72)	NS
Triglycerides, mmol/L ^a^		1.07 (1.53–0.8)	1.08 (0.80–1.46)	1.16 (0.86–1.66)	1.29 (0.87–1.86)	NS
hs-CRP, mg/L ^a^		2.63 (1.30–6)	2.49 (0.93–7.77)	2.10 (1.05–6.34)	3.24 (0.95–8.80)	NS
**Physical activity** (MET-min/wk.) ^a^	297					
MET-score, Total		396 (0–900)	396 (99–1215)	396 (66–792)	297 (0–792)	NS
Vigorous activity ^d^		0 (0–2880)	0 (0–2520)	0 (0–4800)	0 (0–3840)	NS
Moderate Activity ^d^		0 (0–2160)	0 (0–1680)	0 (0–2400)	0 (0–1440)	NS
Walking Activity		148.50 (0–495)	396 (99–792)	272.25 (0–594)	165 (0–495)	0.067
**Food consumption score ^a^**	297					
Total Meat Score		8.80 (5.25–14.90)	11.45 (6.25–17.70)	11.60 (6.45–15.90)	13 (6.6–20.45)	NS
Red Meat		3.45 (1.45–5.90)	4 (1.35–8)	3.35 (1.45–6.35)	5.25 (2.80–9.35) ^1,3^	0.014
White meat		5.45 (3.45–10.75)	6.35 (4.15–10.35)	6.80 (3.45–10.25)	6 (3.80–11)	NS
Seafood		1.35 (0.45–2.45)	1.35 (0.45–2)	1.00 (0.45–3)	1.35 (0.45–2.35)	NS
Fibre score		25.4 (10.35–42.35)	22.95 (12.95–38)	26.73 (18–41.90)	25.50 (13.80–40)	NS
Dairy score		11.9 (6.45–39.25)	15 (7.35–37.30)	26.5 (8–46)	17.75 (8.35–37)	NS
Egg score		2.00 (1.35–6)	2.35 (1–6)	4.00 (1.35–6.45)	4 (1.8–6)	NS

BMI: body mass index; FPG, fasting plasma glucose; HbA1c, glycated haemoglobin; LDL-C, low-density lipoprotein cholesterol; HDL, high-density lipoprotein; CRP, C-reactive protein; TMAO, trimethylamine N-oxide. ^a^ Data are presented as median (IQR (25th–75th)). Kruskal-Wallis test was used in the comparison. ^b^ Data are presented as mean ± standard deviation. One-way ANOVA was used in the comparison. ^c^ Statistically significant association (*p* < 0.05), NS: not statistically significant correlation (*p* > 0.05). ^d^ Data are presented as median (min-max) because the IQR (25th–75th) is zero. ^1^ Q4 is significantly different than Q1 with *p* < 0.05. ^2^ Q4 is significantly different than Q2 with *p* < 0.05. ^3^ Q4 is significantly different than Q3 with *p* < 0.05. ^4^ Q3 is significantly different than Q2, *p* < 0.05.

**Table 3:** Multivariate analysis to assess the relationships between plasma TMAO and markers of metabolic health ^a^.

Variables	R	*p* Value ^b^
Age	0.322	<0.001
BMI	0.054	NS
FPG	0.131	0.024
HbA1c	0.227	<0.001
Cholesterol	0.029	NS
LDL-C	−0.054	NS
HDL-C	0.018	NS
Triglyceride	0.115	0.048
Hs-CRP	0.030	NS

BMI: body mass index; FPG, fasting plasma glucose; HbA1c, haemoglobin A1c; LDL-C, low-density lipoprotein cholesterol; HDL, high-density lipoprotein; CRP, c-reactive protein; r, correlation coefficient. ^a^ Spearman rank correlation to assess the relationships between trimethylamine N-oxide (TMAO) and markers of metabolic health. ^b^ Statistically significant association (*p* < 0.05), NS: not statistically significant correlation (*p* > 0.05).

**Table 4:** Association between plasma TMAO levels and Type 2 diabetes in multiple regression models.

	Model 1			Model 2			Model 3		
Outcome—Type 2 Diabetes	OR	*p* Value	95% CI	OR	*p* Value	95% CI	OR	*p* Value	95% CI
Continuous TMAO	1.11	<0.001 *	1.05, 1.17	0.06	0.04 *	1.00, 1.14	1.05	0.20	0.98, 1.13
Quartiles of TMAO									
Q1 (<2.37)	1 (Reference)			1 (Reference)			1 (Reference)		
Q2 (2.38–3.58)	1.41	0.32	0.71, 2.81	0.98	0.96	0.39, 2.43	1.14	0.79	0.44, 2.93
Q3 (3.59–6.39)	1.64	0.15	0.83, 3.24	0.89	0.80	0.37, 2.16	0.96	0.92	0.39, 2.37
Q4 (>6.40)	6.19	<0.001 *	3.05,12.57	2.81	0.03 *	1.09, 7.27	3.36	0.02 *	1.26, 9.04

Model 1 is non-adjusted. Model 2 adjusted for age, BMI, and level of education. Model 3 adjusted for all of these variables + physical activity MET score and diet (red meat, eggs). * Statistically significant association (*p* < 0.05).

## Data Availability

The datasets used and analysed during the current study are available by contacting the corresponding author upon request.

## Supplementary Materials

The following supporting information can be downloaded at: https://www.mdpi.com/article/10.3390/nu14102093/s1: Figure S1: Flowchart of study participants screening and quantification; Figure S2: Tandem mass spectrometry spectrum of extracted positive ion from trimethylamine *N*-oxide (TMAO) and deuterated stable isotope (d9-TMAO); Table S1: Summary of consumption frequencies of different food items among healthy controls and Type 2 diabetes cases.
