# Supplementary material for: Association between Plasma Trimethylamine N-Oxide Levels and Type 2 Diabetes: A Case Control Study
Source: Nutrients. 2022 May 17;14(10):2093. doi: 10.3390/nu14102093 (PMC9148165; doi:10.3390/nu14102093)
Supplement: Supplementary file 1 [file nutrients-14-02093-s001.zip › nutrients-1702501-supplementary.pdf]

Supplementary Material

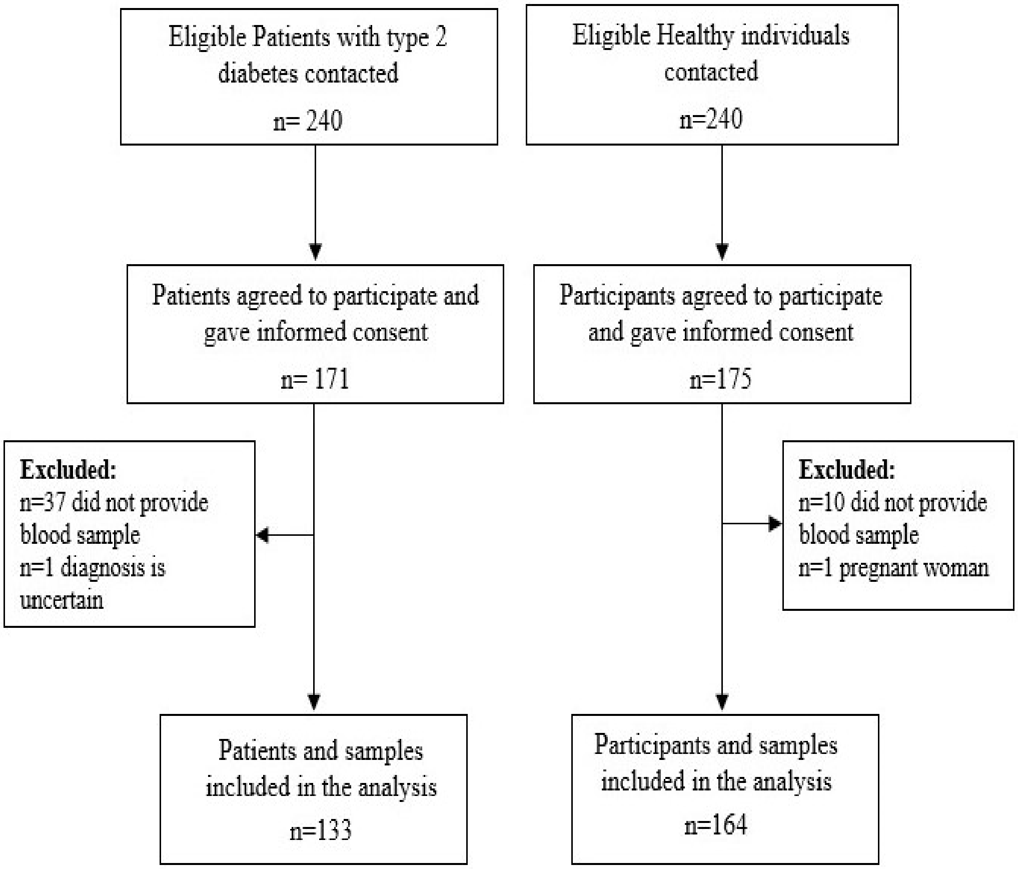

Figure S1. Flowchart of study participants screening and quantification.

a) TMAO

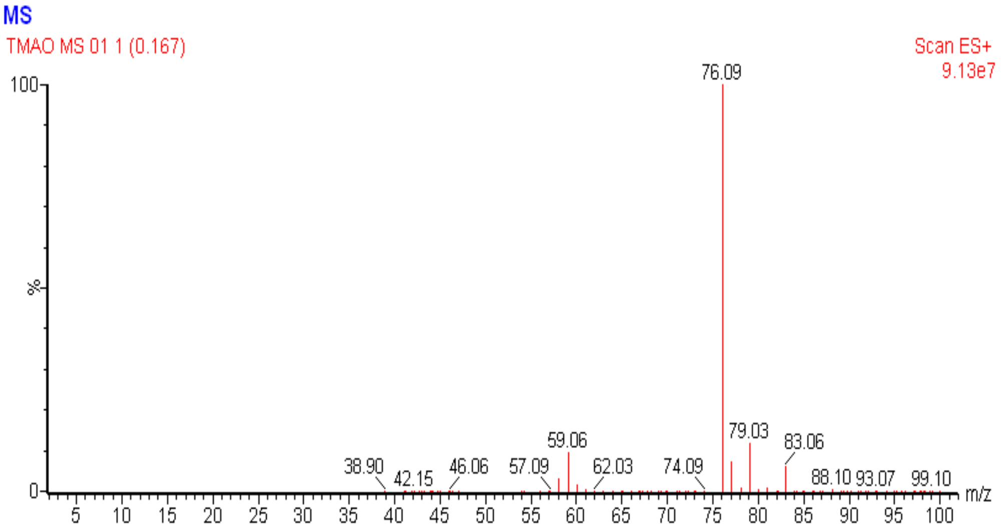

b) d9 (TMAO)

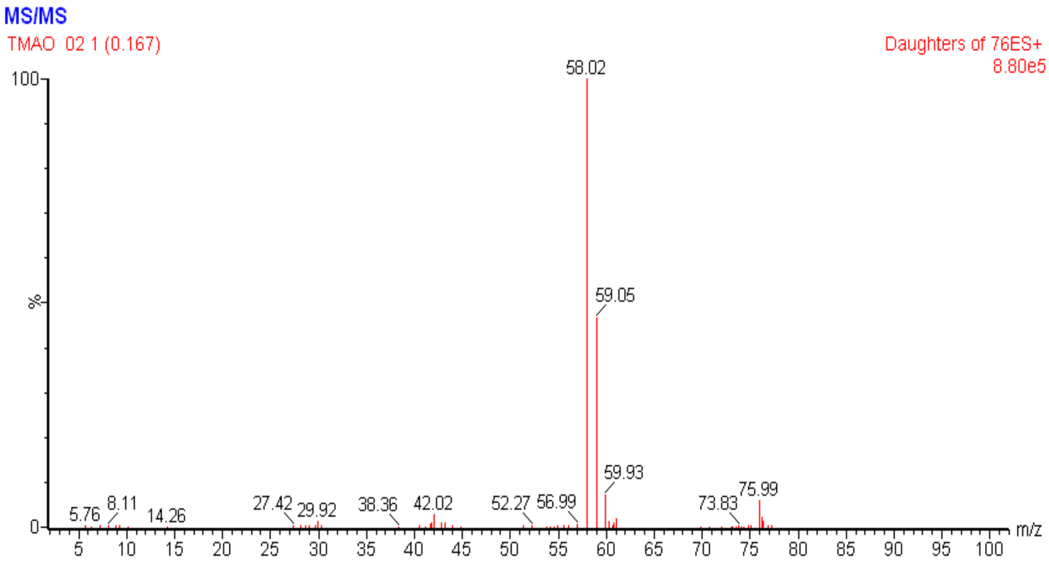

Figure S2. Tandem mass spectrometry spectrum of extracted positive ion from trimethylamine *N*-oxide (TMAO) and deuterated stable isotope (d9-TMAO)

Table S1. Summary of consumption frequencies of different food items among healthy controls and type 2 diabetes cases

	Type 2 diabetes		
	No (n= 164)	Yes (n= 133)	*P*-value [c]
Food Categories [a]			
**Meats**			
Meat Kabsa*	78 (47.5)	84 (63.1)	0.023
Meat stew*	47 (28.6)	71 (53.4)	<0.001
Meat kabab	17 (10.4)	21 (15.8)	NS
Grilled meat	20 (12.2)	23 (17.3)	NS
Chicken Kabsa*	106 (64.6)	103 (77.4)	NS
Chicken stew*	48 (29.3)	74 (55.6)	<0.001
Grilled chicken	73 (44.5)	93 (70)	NS
Chicken kabab	13 (7.9)	25 (18.8)	0.035
Fried Chicken	26 (15.8)	16 (12)	NS
Camel Meat	12 (7.3)	34 (25.5)	<0.001
Fried fish	21 (12.8)	28 (21)	NS
Grilled fish	24 (14.6)	30 (22.5)	NS
Shrimp	19 (11.6)	15 (11.3)	NS
Canned tuna	40 (24.4)	34 (25.5)	NS
Liver	6 (3.7)	9 (6.8)	NS
**Breads And Cereals**			
Wheat Bread	112 (69.3)	104 (78.2)	<0.001
White bread	128 (78)	55 (41.4)	<0.001
Multigrain bread	56 (34.1)	55 (41.4)	NS
White Rice	128 (78)	110 (82.7)	<0.10
Pasta	78 (47.6)	67 (50.4)	NS
cereals	29 (17.7)	15 (11.3)	NS
Boiled potatoes	30 (18.3)	56 (42.1)	<0.001
Fried potatoes	66 (40.2)	36 (27.1)	<0.001
**Sandwiches And Burgers**			
Falafel Sandwich	25 (15.2)	25 (18.8)	NS
Shawerma Sandwich	43 (26.2)	24 (18)	<0.001
Beef Burger	34 (20.7)	9 (6.8)	<0.001
Chicken Burger	32 (19.5)	12 (9)	<0.023
Fried egg sandwich	63 (48.4)	68 (51.1)	0.033
Boiled egg sandwich	48 (29.3)	83 (62.4)	<0.001
Liver sandwich	6 (3.7)	10 (7.5)	NS
Beef sandwich	6 (3.7)	11 (8.3)	NS
**Eggs and Dairy Products**			
Fried Eggs	55 (33.5)	56 (42.1)	NS
Boiled Eggs	40 (24.4)	56 (42.1)	<0.001
Cream cheese	54 (32.9)	39 (29.3)	0.048
White Cheese	88 (53.7)	80 (60.2)	NS
Low fat cheese	60 (36.6)	77 (57.9)	0.03
Full Fat Milk	72 (43.9)	47 (35.3)	0.037
Low fat milk	38 (23.2)	61 (45.9)	<0.001

Full fat Yogurt	52 (31.7)	50 (37.6)	0.016
Low fat yogurt	60 (36.6)	74 (55.6)	0.01
Full fat Laban*	52 (31.7)	42 (31.6)	NS
Low fat Laban	23 (14)	47 (35.3)	<0.001
Full fat cream	20 (12.2)	13 (9.8)	0.034
low fat cream	12 (7.3)	14 (10.5)	NS
**Sweets And Snacks**			
Vegetable Soup	81 (49.4)	103 (77.44)	<0.001
Sugar	70 (42.7)	52 (39.1)	<0.001
Nuts	76 (46.3)	70 (52.6)	NS
Pickles	27 (16.4)	23 (17.3)	NS
**Drinks**			
Black Tea	119 (72.6)	106 (79.7)	<.001
Arabic Coffee	135 (82.3)	114 (85.7)	0.01
Fresh Juice	55 (33.5)	67 (50.4)	0.026
**Fruits**			
Dates	131 (79.9)	121 (91)	<0.001
Apples	82 (50)	97 (73)	<0.001
Pear	39 (23.8)	69 (51.9)	<0.001
Oranges	91 (55.5)	107 (80.5)	<0.002
Banana	94 (57.3)	94 (70.7)	NS
Strawberry	49 (29.9)	34 (25.6)	0.03
Berries	25 (15.2)	22 (16.5)	NS
Mango	17 (10.4)	22 (16.5)	NS
Dried fruits	34 (20.7)	22 (16.5)	0.03
**Vegetables**			
Salad	132 (80.5)	122 (91.7)	<0.001
Onion	103 (62.8)	115 (86.5)	0.03
Garlic	96 (58.5)	105 (79)	0.03
Lentil	27 (16.5)	65 (48.9)	<0.001
Beans	53 (32.3)	72 (54.1)	0.03
Broccoli	30 (18.3)	45 (33.8)	0.05
Corn	40 (24.4)	53 (39.9)	<0.001
Pumpkin	51 (31.1)	79 (59.4)	<0.001
Spinach	25 (15.2)	52 (39)	<0.001
Cauliflower	40 (24.4)	67 (50.4)	<0.001
Oils and Meat Fat			
Olive oil	134 (81.7)	99 (74.4)	NS
Vegetable oil	133 (82)	107 (80.5)	NS
Butter	24 (14.6)	24 (18)	NS
Margarine	11 (6.7)	19 (14.3)	0.031
**Meat fat**			
Eat most	4 (2.4)	8 (6)	NS
Eat little	32 (19.5)	11 (8.3)	0.019
Food Groups Scores [b]			
Total Meat	8.35 (5.35-14.57))	14.20 (8.25-19.80)	<0.001
Red meat	3.58 (1.35-6.05)	4.45 (2.25-8.90)	0.042
White meat	5.35 (3.80-10.13)	7.45 (3.45 -11)	NS

Seafood	1 (0.45-2.45)	1.35 (0.45-2.35)	NS
Eggs	2 (1 - 4.95)	4 (1.90 - 8)	<0.001
Dairy Products	12.73 (7.23-31.83)	21.45 (9.85-53.40)	0.023
Fibers[#]	21.90 (12.45-37.90)	29.50 (19.95-44.95)	0.021

[a] Data are expressed as n (%) representing the frequency and proportion of participants who reported consuming items at least once weekly or more.

[b] Data are presented as median (IQR (25th-75th))

[c] Statistically significant association ($p<0.05$), NS: not statistically significant correlation ($p>0.05$)

* Food recipes: Kabsa, traditional food consist of meat, rice and species; Stew: consists of cuts of meat, broth and vegetables; Laban: is a watered down yogurt

[#] Fibers score does not account for fibers from fruits and vegetables.
